# Mental health problems in adolescents with cochlear implants: peer problems persist after controlling for additional handicaps

**DOI:** 10.3389/fpsyg.2015.00953

**Published:** 2015-07-15

**Authors:** Maria Huber, Thorsten Burger, Angelika Illg, Silke Kunze, Alexandros Giourgas, Ludwig Braun, Stefanie Kröger, Andreas Nickisch, Gerhard Rasp, Andreas Becker, Annerose Keilmann

**Affiliations:** ^1^Department of Otorhinolaryngology, Head and Neck Surgery, Paracelsus Medical University SalzburgSalzburg, Austria; ^2^Department of Otorhinolaryngology, Cochlear Implant Center Freiburg, University of FreiburgFreiburg, Germany; ^3^Department of Otolaryngology, Hannover Medical SchoolHannover, Germany; ^4^Socialpediatric Center MunichMunich, Germany; ^5^Section of Communication Disorders, Clinic of Otorhinolaryngology, Head and Neck Surgery, University of MainzMainz, Germany; ^6^Department of Child and Adolescent Psychiatry, University of GoettingenGoettingen, Germany

**Keywords:** cochlear implants, adolescents, hearing loss, multi handicap, mental health problems, SDQ, peer problems, multi-center study

## Abstract

The aims of the present multi-center study were to investigate the extent of mental health problems in adolescents with a hearing loss and cochlear implants (CIs) in comparison to normal hearing (NH) peers and to investigate possible relations between the extent of mental health problems of young CI users and hearing variables, such as age at implantation, or functional gain of CI. The survey included 140 adolescents with CI (mean age = 14.7, *SD* = 1.5 years) and 140 NH adolescents (mean age = 14.8, *SD* = 1.4 years), their parents and teachers. Participants were matched by age, gender and social background. Within the CI group, 35 adolescents were identified as “risk cases” due to possible and manifest additional handicaps, and 11 adolescents were non-classifiable. Mental health problems were assessed with the Strengths and Difficulties Questionnaire (SDQ) in the versions “Self,” “Parent,” and “Teacher.” The CI group showed significantly more “Peer Problems” than the NH group. When the CI group was split into a “risk-group” (35 “risk cases” and 11 non-classifiable persons) and a “non-risk group” (*n* = 94), increased peer problems were perceived in both CI subgroups by adolescents themselves. However, no further differences between the CI non-risk group and the NH group were observed in any rater. The CI risk-group showed significantly more hyperactivity compared to the NH group and more hyperactivity and conduct problems compared to the CI non-risk group. Cluster analyses confirmed that there were significantly more adolescents with high problems in the CI risk-group compared to the CI non-risk group and the NH group. Adolescents with CI, who were able to understand speech in noise had significantly less difficulties compared to constricted CI users. Parents, teachers, and clinicians should be aware that CI users with additionally special needs may have mental health problems. However, peer problems were also experienced by CI adolescents without additional handicaps.

## Introduction

A cochlear implant (CI) is a prosthesis for the hair cells in the inner ear of persons with severe or profound hearing loss. In children with a severe or profound sensorineural hearing loss it usually allows the development of speech understanding and speech production. Long-term studies have however shown that language and speech performance improve slowly over time after cochlear implantation and require years to reach the final level (Beadle et al., 2005; Uziel et al., 2007). Therefore, the age at which children receive their first CI is one of the strongest predictors of hearing and speech skills after cochlear implantation (Nikolopoulos et al., 1999; Sharma et al., 2002; Lesinski-Schiedat et al., 2004; Connor et al., 2006). The language development of children implanted at a very young (<2 years) age is very similar to that of their normal hearing (NH) peers (Spencer et al., 2004; Uziel et al., 2007).

In the last years further areas gained importance in CI research beyond hearing and speech of children and adolescents growing up with CIs. These include academic performance (e.g., Beadle et al., 2005; Uziel et al., 2007; Huber et al., 2014), cognition (e.g., Soleymani et al., 2014) and health related quality of life (e.g., Loy et al., 2010). Most recently, mental health problems^1^ of children and adolescents with CIs gained attention (Hintermair, 2007; Dammeyer, 2010; Huber and Kipman, 2011; Theunissen et al., 2011, 2012, 2013; Anmyr et al., 2012).

Studies about young persons with a hearing loss and without a CI indicate that unsatisfactory progress in speech- and language development (Barker et al., 2009; Stevenson et al., 2010) and/or communication problems (Hogan et al., 2011) promote mental health problems, whereas speech intelligibility protects against mental health problems (Polat, 2003). Cochlear implantation enables the development of language and speech (Nikolopoulos et al., 1999; Sharma et al., 2002; Lesinski-Schiedat et al., 2004; Spencer et al., 2004; Beadle et al., 2005; Connor et al., 2006; Uziel et al., 2007). Therefore, we expect that in the long term cochlear implantation has a positive effect on the mental health of children and adolescents with a hearing loss.

Furthermore, hearing variables like the age at first and second CI and functional gain of the CI(s) (aided thresholds) may be associated with the mental health outcomes of young CI users.

However, the number of studies addressing mental health problems of adolescents with CIs is still limited (compare Table 1).

**Table 1:** **Studies on mental health problems of children older than six years and adolescents with cochlear implants (at least 20% of the study group)**.

**Nr**	**Mean age**	**Mental health instrument**	**Mainstream school**	**Outcome—mental health problems**
[1]	10	SDQ^3^ parents	No information	“… as the prevalence of socioemotional problems in the sample of deaf and hard of hearing children was… greater for almost all scores…” No association with the degree of hearing loss of “the threes groups <70 dB, 70–90 dB, >90 dB.”
[2]	13	SDQ teacher	100% in schools for persons with hearing loss	3.7 times more “psychosocial difficulties” compared to normally hearing peers. Persons with additional disabilities have 3 times more mental health problems compared to persons without. No association with the degree of hearing loss.
[3]	15	SDQ self SDQ parents SDQ teacher	75%	Significantly more peer problems in the CI-group than in the comparison group of normally hearing peers. Apart from that there was no significant difference between CI- and normal hearing group. Pupils of schools for persons with hearing loss and sign language competent persons showed more problems. The better the speech perception outcomes and reading-speech comprehension, the less are the mental health problems.
[4]	9–15	SDQ self SDQ parents SDQ teacher	29%	Children rated significantly more mental health problems than parents and teachers did.
[5]	11	(a)^4^,(b)^5^	CI (59%) HA (64%)	“Hearing impaired children reported more depressive symptoms than normally hearing children.”
[6]	12	(c)^6^, (d)^7^, (e)^8^ Intelligence- and language tests	CI (53%) HA (59%)	“Levels of anxiety in children with cochlear implants and normally hearing children were similar.” Children with HA showed higher level of social anxiety. “Early implantation was associated with lower levels of ….anxiety.”
[7]	12	(f)^9^, (g)^10^, (h)^11^, parts of (i)^12^ Intelligence- and language tests	CI (60%) HA (60%)	“More behavioral problems occurred in HI than in NH children.” More problems were shown for pupils of schools of the deaf, higher age, males, disadvantages in social background, lower IQ, and delayed language development. No association with degree of hearing loss or aided threshold was found.

*[1] Hintermair (2007), [2] Dammeyer (2010), [3] Huber and Kipman (2011), [4] Anmyr et al. (2012), [5] Theunissen et al. (2011), [6] Theunissen et al. (2012) [7] Theunissen et al. (2013)*.

Hintermair (2007) investigated the “prevalence of socioemotional problems” in 213 children and adolescents with hearing loss, including 50 children/adolescents with CIs. However, this paper did not explicitly inform about the specific prevalence rates of the CI group. For more information see Table 1.

Dammeyer (2010) found no significant differences in psychosocial development between 119 “deaf” participants, 116 “hard of hearing” participants and 92 participants with CIs^2^. However, the authors noted, that the sample of children with CIs was non-representative. Furthermore, NH children were not included as a control group in this study. For more information see Table 1.

To the best of our knowledge, only very few studies so far included a NH comparison group when assessing the mental health problems of children or adolescents with CIs.

Huber and Kipman (2011) compared mental health problems between 32 adolescents with CIs and 212 NH peers. Adolescents with CIs have more peer problems compared to their NH peers. But no further differences in other domains, including emotional problems, conduct problems, hyperactivity or prosocial behavior were observed between CI group and normally hearing group. The CI group was however small and there was no matching between CI- and NH group. For more information see Table 1.

Theunissen et al. (2011) examined levels of depressive symptoms in 56 children with hearing aids (with a hearing loss reaching from moderate to profound), 27 children with cochlear implants and 117 NH children. However, the CI group was small. The paper did not inform about the specific prevalence rates of the CI group and there was no matching between control and study group. For more information see Table 1.

Additionally, Theunissen et al. (2012) investigated levels of anxiety in 51 children with hearing aids (with a hearing loss reaching from moderate to profound), 32 children with cochlear implants and 127 children “without hearing loss.” The CI group was however small and there was no matching between CI- and NH group. For more information see Table 1.

Furthermore, Theunissen et al. (2013) compared behavioral problems (aggression, delinquency, oppositional behavior, psychopathy, and attention deficit and hyperactivity disorder) between 75 children and adolescents with hearing aids (with a hearing loss, reaching from moderate to profound), 57 children and adolescents with a CI, and 129 NH peers. CI users showed less behavioral problems than children with hearing aids. However, the paper did also not inform about the specific prevalence rates of the CI group and there was no matching between control and study group. For more information see Table 1.

Therefore, the question, whether cochlear implantation can protect children and adolescents with a hearing loss against mental health problems requires further attention. Particularly, more closely controlled studies comparing young CI users to NH peers are needed to evaluate whether the prevalence rate of mental health problems is still higher in children with CI compared to NH children.

Despite a matching for age, gender and social background, one should also consider that the risk for additional disabling health conditions is increased in the population of persons, who grow up with a hearing loss.

According to the American Academy of Pediatrics, American Academy of Pediatrics, Joint Committee on Infant Hearing (2007), 30–40% of all US children with a hearing loss are suffering from additional disabling health conditions, such as genetic disorders, infections, e.g., meningitis, or conditions as consequences of critical events, e.g., maternal rubella or preterm birth. These conditions are not only associated with hearing loss, but also with brain pathologies, neurological disorders, physical handicaps, borderline or subnormal IQ, and visual impairment. According to the Gallaudet Research Institute (2011), 29% of all US- children and adolescents with a hearing loss are suffering from additional disabilities or handicaps (“legal blindness,” developmental delay, learning disability, traumatic brain injury, mental retardation, Autism, Usher syndrome).

It should be noted, that disabling health conditions may also have a negative effect on the mental health of the individuals. Both, children with a hearing loss (Van Eldik, 2005; Van Gent et al., 2007) as well as NH children (Carvill, 2001; Barkauskiene and Bieliauskaite, 2002; Dekker et al., 2002; Leask et al., 2002; Glazebrook et al., 2003; Hemmings et al., 2006; Kaptein et al., 2008; Emerson et al., 2010; Backenson et al., 2013) are concerned. All persons with disabling health conditions, such as visual impairment (Carvill, 2001), intellectual disabilities or subnormal IQ (Carvill, 2001; Dekker et al., 2002; Van Eldik, 2005; Hemmings et al., 2006; Van Gent et al., 2007; Kaptein et al., 2008; Emerson et al., 2010), learning disabilities (Barkauskiene and Bieliauskaite, 2002; Emerson et al., 2010; Backenson et al., 2013), brain disorders (Glazebrook et al., 2003), childhood infections and neurological soft signs (Leask et al., 2002) show an increased risk for mental health problems and disorders. It is of interest, whether potential mental health problems in children with CIs can be related to these additional risk factors rather than the hearing loss *per se*.

In the case of CIs it has to be additionally taken into account that some children, e.g., those with Mondini Dysplasia, have congenital malformations of the inner ear, which complicates the cochlea implantation (Aschendorff et al., 2009). We assume that this group is also at risk for mental health problems, since language- and speech outcomes are variable in young CI users with these complications (Aschendorff et al., 2009).

To address these questions, we initiated a multi-center study assessing mental health problems in a large sample of 140 adolescents with CIs, who were closely matched to 140 NH adolescents for age, gender and social background.

The aim of the study was to investigate, whether more mental health problems were prevalent in adolescents with CIs than in their NH peers. We hypothesize that differences in mental health problems between CI users and NH peers are attributable to CI users with additional handicaps (intellectual disabilities or learning disorders, visual impairments or with inner ear malformations) rather than CI users without additional handicaps. A further aim was to provide information about the relation of hearing variables (e.g., age at cochlea implantation, functional gain of the CI of the better ear, i.e., aided thresholds, ability to understand in noise, use of hearing aids before implantation/minimal benefit of hearing aid prior to implant) to the mental health of CI users (see also corresponding information on Hintermair, 2007; Dammeyer, 2010; Theunissen et al., 2012, 2013 in Table 1).

## Methods

This study was conducted as a multi-center study. The centers Cochlear Implant Center Freiburg, University of Freiburg, Hannover Medical School, Department of Otolaryngology Hannover, University Medical Center, University Mainz, Socialpediatric Center Munich, and Cochlear Implant Center, University Clinic Salzburg participated in the study.

### Participants

The study group was comprised of 140 adolescents with CIs (68 boys, 72 girls) and their hearing parents and teachers, 30 from Freiburg, 43 from Hannover, 44 from Mainz and 23 from Munich (see demographic data in Tables 2, 3). Our response rate was 79% out of 178 possible cases^13^.

**Table 2:** **Demographic data of 140 adolescents with cochlear implants participating in the study including (“non-risk group”) 46 CI users with indication for additional handicaps and non-classifiable persons (“risk group”) and 94 CI users without additional handicaps (“non-risk group”)**.

	**All**	**Risk**	**Non-risk**
Girls, number (percent)	68 (49)	18 (39)	50 (53)
Boys, number (percent)	72 (51)	28 (61)	44 (47)
Age (years): mean (*SD)*	14.72 (1.51)	14.68 (1.56)	14.74 (1.49)
Causes of deafness, numbers (percent)			
Meningitis	8 (6)	8 (17)	0
Rubella	2 (1)	2 (4)	0
CMV	5 (4)	5 (11)	0
Otitis media	2 (1)	0	2 (2)
Waardenburg syndrome	2 (1)	2 (4)	0
Mondini Dysplasia	3 (2)	3 (7)	0
“Genetic” (non syndromal)	11 (8)	8 (17)	3 (3)
Other diseases and reasons	11(8)	0	11 (12)
Unknown	96 (69)	21 (46)	75 (80)
Age at first fitting of hearing aids (months): mean (*SD*) *n* = 60	20.23 (15.65)	20.93 (14.03)	20.02 (16.26)
Benefit of hearing aids (minimal perception of acoustic stimuli with hearing aids) prior to implant, number (percent)^*^	72 (53)	25 (57)	47 (51)
Age (years) at 1st implantation: mean (*SD*)	4.53 (3.95)	4.65 (3.91)	4.47 (3.99)
Duration (years) of 1st implants use: mean (*SD*)	9.99 (3.86)	9.87 (3.75)	10.05 (3.93)
Unilateral cochlear implantation, number (percent)	72 (51)	30 (65)	41 (45)
Bilateral cochlear implantation, number (percent)	68 (49)	16 (35)	51 (55)
Age (years) at 2nd implantation: mean (*SD*) *n* = 68	10.05 (3.30)	9.92 (4.05)	10.10 (3.08)
Inter-implant interval, years: mean (*SD*) *n* = 68	4.41 (2.72)	4.65 (3.39)	4.33 (2.50)
Duration (years) of 2nd implant use: mean (*SD*) *n* = 68	7.05 (3.78)	5.67 (4.43)	7.49 (3.48)
Audiogramm (aided treshold): 500 Hz/1000/2000 k/4000 kHz	30.3/30.0/30.5/33.0	30.4/30.5/32.2/33.5	30.3/29.8/29.8/32.9
Is understanding in noise, number (percent)^†^	88 (73)	25 (66)	63 (77)
Speech perception ^‡^(%)Monosyllables (60dB): mean (*SD*) n too small	–	–	–
Monosyllables (65dB): mean (*SD*) *n* = 106.71/37	66 (23)	63 (24)	28 (23)
Monosyllables (70dB): mean (*SD*) *n* = 31.22/8	74 (25)	64 (30)	77 (23)
Primary mainstream school, number (*percent*)	43 (32)	9 (20)	34 (37)
Primary school for persons with hearing loss, number (percent)	88 (65)	34 (76)	54 (59)
Other primary schools, number (percent)	4 (3)	2 (4)	2 (2)
Secondary mainstream schools, number *(percent)*	58 (41)	15 (33)	43 (46)
Secondary school for persons with hearing loss, number (percent)	82 (59)	31 (67)	51 (54)

* *According to the rating of the parents (4 point rating scale: 1 = some profit, 4 = no profit at all)*.

† *Evaluated by the audiologists 0 = is understanding 1 = is not understanding*.

‡ *In quiet*.

**Table 3:** **Educational level and employment skills of** ***n*** **= 136 parents of CI users including 46 CI users with indication for additional handicaps and non-classifiable persons (“risk group”) and 94 CI users without additional handicaps (“non-risk group”) and** ***n*** **= 60 parents of normally hearing peers (matched by age, gender, and social background)**.

	**CI**			**Hearing**
	**All CI**	**Risk**	**Non-risk**	
	**N (%)**			**N (%)**
**EDUCATIONAL LEVEL FATHER**				
Secondary school	39 (31)	10 (26)	29 (33)	17 (28)
Vocational school	36 (29)	11 (28)	25 (29)	19 (32)
Grammar school	19 (15)	8 (21)	11 (13)	11 (18)
College or University	27 (21)	8 (21)	19 (22)	13 (22)
No secondary mainstream qualification	5 (4)	2 (5)	3 (3)	/
EMPLOYMENT SKILLS^*^ FATHER				
0	0	0	0	3 (6)
1	15 (12)	4 (10)	11 (13)	3 (6)
2	92 (72)	32 (78)	60 (69)	34 (63)
3	21 (16)	5 (12)	16 (18)	13 (24)
**EDUCATIONAL LEVEL MOTHER**				
Secondary school	39 (29)	14 (32)	25 (27)	18 (30)
Vocational school	61 (45)	20 (46)	41 (45)	19 (31)
Grammar school	12 (9)	4 (9)	8 (9)	14 (23)
College or University	14 (10)	3 (7)	11 (12)	10 (16)
No secondary mainstream qualification	10 (7)	3 (7)	7 (8)	/
EMPLOYMENT SKILLS^*^ MOTHER				
0	13 (10)	4 (10)	9 (10)	10 (18)
1	22 (18)	14 (33)	8 (10)	4 (7)
2	80 (64)	22 (52)	58 (70)	35 (61)
3	10 (8)	2 (5)	8 (10)	7 (12)

*Key for employment skills: 1 = unskilled work, 2 = jobs demanding vocational/training qualifications up to college level, 3 = jobs demanding college/university degrees, 0 = others*.

* Orientation ISCO 88 International Standard Classification of Occupation (International Labor Office)^14^^*^The higher the number the higher the parents' ISCO-Level.

All adolescents of the study group were between 12 and 17 years old (mean age = 14.72 years, *SD* = 1.51 years), were diagnosed with severe or profound hearing loss before the age of 24 months and had been using their first CI for at least three years.

In 35 adolescents of the study group we found indications for additional handicaps. These “risk cases” fulfilled at least one of the following criteria: (i) risk for general learning disorder (borderline intellectual functioning) or intellectual disability (31 cases), (ii) visual impairment (1 case), or (iii) inner ear malformations (3 cases). 94 CI users had no additional handicaps, and 11 CI users could not be clearly assigned (see Procedures and Table 2 for further information).

In 21 cases the risk could clearly be attributed to the respective etiology for hearing loss of the young CI users. 17 cases out of the 21 fulfilled criterion (i): 5 cases with CMV, two cases with Rubella, one case with Dystonia, one case with Toxoplasmosis, and 8 cases with Meningitis. One case (out of the 21) with Usher syndrome fulfilled criterion (ii) and 3 cases with Mondini dyplasia fulfilled criterion (iii). In 14 of 35 cases the risk could not or not clearly be associated with the etiology of the hearing loss. All 14 cases met criterion (i). In 9 out of these 14 cases clinical files indicated a distinct developmental delay. In 4 cases clinical files informed about additional conditions (e.g., some prenatal infections) with suspicion for intellectual disabilities, in one case the file informed about a neurological condition. Despite these risk factors in the etiology of the hearing loss, risk cases and non-risk cases did not differ in any demographic or hearing variables as summarized in Tables 2, 3.

The comparison group consisted of 140 normally hearing adolescents (68 boys and 72 girls, mean age = 14.8 years, *SD* = 1.4 years) without any intellectual or visual impairments, their hearing parents and teachers. This group was selected from a pool of 212 Salzburgian normal hearing adolescents as described elsewhere [13]. A 1-to-1 matching procedure was employed to match each adolescent with CI to a normally hearing peer of the same sex and comparable age and social background. Social background data are shown in Table 3. All adolescents in the control group were enrolled in mainstream education programs.

### Instruments

Mental health problems were assessed with the “Strengths and Difficulties Questionnaire” (SDQ^15^) (Goodman, 1997). The SDQ evaluates emotional, behavioral and social problems of children and adolescents aged about 3–17 years. It can also be used as screening measure for mental health disorders, which was not the case in the present study. Its good psychometric properties have been confirmed by many studies worldwide (Goodman and Scott, 1999; Koskelainen et al., 2000; Goodman et al., 2003; Meltzer et al., 2003; Muris et al., 2003; Hawes and Dadds, 2004; Woerner et al., 2004; Becker et al., 2004a,b; Du et al., 2008). The brief 25 item rating scale addresses emotional symptoms (ES), hyperactivity-inattention (HA), conduct problems (CP), peer-problems (PP) and pro-social behavior (PBS), (social strengths, e.g., altruism). The scores of ES, HA, CP and PP can be summarized to the “Total Difficulty Score” (TDS). Due to multivariate analysis procedures (see Statistics), TDS was not analyzed further in the present study. SDQ versions are available for parents, teachers and as self ratings for children from 11 years of age and older. There are three response categories; 0 = not true, 1 = somewhat true and 2 = certainly true. For ES, HA, CP, and PP higher values mean more problems, for PBS higher values mean less problems. The SDQ has been translated and validated for the German language (Becker et al., 2004a,b).

### Procedures

The investigation was conducted between January 2012 and January 2013. The participants were recruited on the occasion of the annual appointment in the clinics. Both, adolescents and their parents were asked to participate. In the case of agreement, all adolescents and their parents were surveyed individually. Medical and audiological data were obtained from clinic files. Other demographic data were collected by parental surveys. The patients completed the SDQ questionnaire under surveillance by a clinic member. In 16 cases support was needed, whereby the SDQ questions were given additionally in an adapted format, with standard sentences—following a written guideline-shortened and with paraphrases, presented both orally and written. This support did not replace the original SDQ questionnaire. The use of a sign language interpreter was not required. The parents filled in the questionnaires (SDQ, demographic data) at the same time, however separately. In the case of their agreement, the teachers received the SDQ from the parents and sent it back to the investigators via mail. Teacher ratings were available for 55 adolescents of the study group and 42 adolescents of the control group.

#### Assignment of “risk cases” in the study group

Clinical files reported about cases of visual impairment, criterion (ii), or inner ear malformations, criterion (iii). However, not all clinical files provided data about general learning disorders and intellectual disability, criterion (i). To compensate possible missing data, a pediatric assessment was carried out. Thereby for all adolescents in the study group, a pediatrician evaluated whether the available anonymized data about physical diagnoses, IQ, neurological status, indication for brain disorders (from the clinical files and an anamnesis questionnaire) indicated an additional disability. The pediatrician also assessed for every single case, if the physical diagnoses (e.g., of an infection, a genetic syndrome, primarily as cause for deafness) were associated with an increased incidence of brain disorders and/or neurological disorders, which are possible causes for general learning disabilities or intellectual disabilities.

#### For the recruitment of the comparison (NH) group

We used existing SDQ data and demographic data, collected in two mainstream schools (one secondary school, one secondary grammar school) and one apprenticeship institution in Salzburg (also mainstream education). For economic reasons, all NH adolescents were surveyed in groups, but seated separately, so that neither communication with others nor looking at the questionnaires of other peers was possible. Parents and teachers filled out the questionnaires (SDQ) individually. The parents received the SDQ from the teachers and sent it back to the teacher via mail. The survey of the comparison group was performed anonymously.

### Statistics

Statistical analysis was carried out using IBM SPSS Statistics, version 22. Inter-rater agreements between self-, parent-, and teacher-ratings were assessed for each SDQ scale in adolescents with CI and normal hearing group via Pearson correlations. To compare the correlations of the CI group with the comparison group, Fisher's z-transformations were computed.

To test, whether young CI users were comparable to NH peers in their mental health problems, SDQ ratings were compared between CI group and NH group using multivariate ANOVAs over the four problem areas (ES, CP, HA, PP). As the inter-rater agreement in the NH group was only low and only few teacher ratings were available, the analysis was conducted separately for self-, parent- and teacher-ratings. The significance level was thus Bonferroni corrected to 0.017. To evaluate, in which areas the problems manifested, independent-samples *t*-tests were conducted to compare ratings for each problem area separately. PBS was also compared separately. The multivariate ANOVAs were repeated after splitting the study group into a “risk group” and a “non-risk” group, and *post-hoc* Tukey tests were used to evaluate in which problem areas and between which groups the differences occurred. Additionally, cluster analyses were performed on the 5 scores (ES, CS, HA, PP, PBS) of self-, parent-, and teacher-ratings of the total sample (study group and control group) using the new two-step algorithm implemented in SPSS. Then the distribution of adolescents with CIs and normally hearing adolescents in the resulting clusters was reported. Two-step cluster analysis is an advancement of traditional cluster analysis techniques and has the advantage of being able to deal with multiple scalings of variables, a large data-set and in particular automatically determine the number of clusters in the sample. The algorithm applies a two-step procedure by first pre-clustering data using a sequential approach and second grouping data into sub-clusters using the agglomerative hierarchical clustering method. Thereby the distance between two clusters is defined as the corresponding decrease in log-likelihood by combining them in one cluster.

To test, whether mental health problems of adolescents with CIs were related to hearing variables, scores for each SDQ scale were Pearson-correlated to the following audiological variables: (i) benefit of hearing aid prior to implant, (ii) age at fitting of the 1st CI, (iii) speech discrimination (monosyllables, 65 dB), (iv) ability to hear and to understand speech in noise and (v) functional gain of CI aided threshold (mean over functional gain at thresholds 0.5 kHz, 1 kHz, 2 kHz, 4 kHz). Because of missing data the age at first fitting of HA was not taken into account.

### Ethical approval

The study was approved by the ethics committees in Salzburg (Ethikkommission für das Bundesland Salzburg), Munich (Ethikkommission der LMU München), Mainz (Ethikkommission der Landesärztekammer Rheinland-Pfalz), Freiburg (Ethik-Kommission der Albert-Ludwigs-Universität Freiburg) and Hannover (Ethik-Kommission der MHH).

## Results

### Inter-rater agreement between SDQ self-, parent-, and teacher ratings

Pearson correlations representing inter-rater agreement between self- parent-, and teacher ratings on all SDQ subscales in the CI group and normally hearing group are summarized in Table 4. In the CI group, agreement between self and parent ratings was high on all scales. Agreement between teacher ratings and self- and parent ratings respectively was high on all scales except ES. In the NH group, correlations between self-, parent- and teacher ratings were weak and only few reached statistical significance. Most correlations were significantly stronger in the CI group than in the NH group according to Fisher's z-transformation, indicating higher inter-rater agreement in the CI group than in the NH group.

**Table 4:** **Inter-rater agreement between self, parent and teacher ratings in CI group and NH group: Correlation between SDQ scales from different informants**.

**SDQ scales**	**CI group**			**NH group**			**Comparison**		
	**S × P**	**S × T**	**P × T**	**S × P**	**S × T**	**P × T**	**S × P**	**S × T**	**P × T**
	**(*n* = 128)**	**(*n* = 55)**	**(*n* = 55)**	**(*n* = 68)**	**(*n* = 43)**	**(*n* = 40)**			
Total difficulties	0.51^***^	0.37^**^	0.41^**^	0.15	0.14	0.06	2.69^**^	1.18	1.75^*^
Emotional symptoms	0.45^***^	0.19	0.21	0.24^*^	0.40^**^	0.31^T^	1.57	−1.10	−0.50
Conduct problems	0.48^***^	0.30*	0.39^**^	0.17	0.18	−0.08	2.30^**^	0.61	2.29^**^
Hyperactivity-inattention	0.48^***^	0.44^***^	0.61^***^	0.06	0.04	0.31^T^	3.03^***^	2.06^*^	1.81^*^
Peer problems	0.46^***^	0.28^*^	0.39^**^	0.21^T^	0.30^T^	−0.12	2.86^**^	−0.10	2.48^**^
Prosocial behavior	0.28^***^	0.25^T^	0.35^**^	0.32^**^	0.25	−0.12	−0.29	0.00	2.26^**^

*Comparison of correlations between CI group und NH group*.

* *p < 0.05*,

** *p < 0.01*,

*** *p < 0.001*,

T *p < 0.1; S = self, P = parent, T = teacher*.

*^1^Correlations significantly stronger in the CI group than in the normal hearing group according to Fisher's z-transformation (at least p < 0.05 one-sided)*.

### Comparison of SDQ results between CI group and NH group, matched by age, gender and social background

#### Total group (n = 280, 140 CI users, 140 NH peers)

Table 5 shows the SDQ results of the CI group and the NH group. The higher the SDQ score, the more pronounced are the mental health problems rated. To evaluate, whether mental health problems differed significantly between CI group and NH group, multivariate ANOVAs over the four difficulty areas emotional symptoms (ES), conduct problems (CP), hyperactivity-inattention (HA), peer problems (PP) were conducted for each rater (self/parent/teacher). The significance level was thus Bonferroni corrected to *p* = 0.017. Subsequent univariate analyses (*t*-tests) were performed to evaluate in which area the problems were observed. Multivariate analyses revealed highly significant group differences in SDQ self- [*F*_(4, 263)_ = 4.97, *p* = 0.001], parent- [*F*_(4, 203)_ = 3.46, *p* < 0.01] and teacher ratings [*F*_(4, 92)_ = 4.30, *p* < 0.01]. Mental health problems were rated significantly higher in CI adolescents compared to NH adolescents. Subsequent univariate analyses (compare Table 5 for *t*-values) revealed that these differences were attributable to PP, which were rated significantly higher in CI adolescents compared to NH adolescents by self-, parent- and teacher ratings (self: *t* = 3.68, *p* < 0.001, parents *t* = 2.85, *p* < 0.004, teacher: *t* = 2.52, *p* = 0.01.

**Table 5:** **Scale means (SD) and** ***T*****-values for comparison of SDQ mean scores between CI group (*****n*** **= 129 self,** ***n*** **= 139 parents and** ***n*** **= 55 teachers) and NH group (*****n*** **= 140 self,** ***n*** **= 70 parents and** ***n*** **= 42 teachers), matched by age, gender and social background**.

	**Total difficulties**	**Emotional symptoms**	**Conduct problems**	**Hyperactivity inattention**	**Peer problems**	**Prosocial behavior**
**CI**						
Self	11.6 (4.9)	2.6 (1.9)	2.2 (1.5)	3.8 (1.8)	3.0 (1.8)	7.7 (1.5)
Parents	10.1 (5.6)	2.4 (2.1)	2.0 (1.7)	3.1 (2.3)	2.6 (2.1)	8.0 (1.8)
Teachers	8.0 (6.6)	2.3 (2.5)	0.9 (1.5)	2.2 (2.6)	2.5 (2.7)	7.4 (2.2)
**HEARING**						
Self	10.5 (5.0)	2.6 (2.1)	2.2 (1.7)	3.5 (1.9)	2.2 (1.7)	8.0 (1.5)
Parents	8.7 (5.9)	2.0 (2.0)	2.1 (1.8)	2.8 (2.4)	1.8 (1.9)	7.7 (2.2)
Teachers	6.3 (4.9)	1.4 (1.6)	1.5 (1.9)	2.1 (2.0)	1.3 (2.0)	7.6 (2.6)
t-Self	1.85	−0.32	0.35	1.73	3.68^***^	−1.40
t-Parents	1.59	1.22	−0.70	0.86	2.85^**^	1.10
t-Teachers	1.38	2.13	−1.79	0.30	2.52^*^	−0.46

*Higher scores indicate more problems except for prosocial behavior (lower scores indicate more problems)*.

* *p < 0.05*,

** *p < 0.01*,

*** *p < 0.001*.

Self-, parent-, and teacher-ratings did not differ significantly in any other difficulty area (ES, CP and HA). Prosocial behavior (PBS) ratings of any rater did also not differ between CI children and NH children (compare Table 5).

#### Split CI group: comparison of CI risk group (*n* = 46), CI non-risk group (*n* = 94) and NH group (*n* = 140)

**Figure 1:**
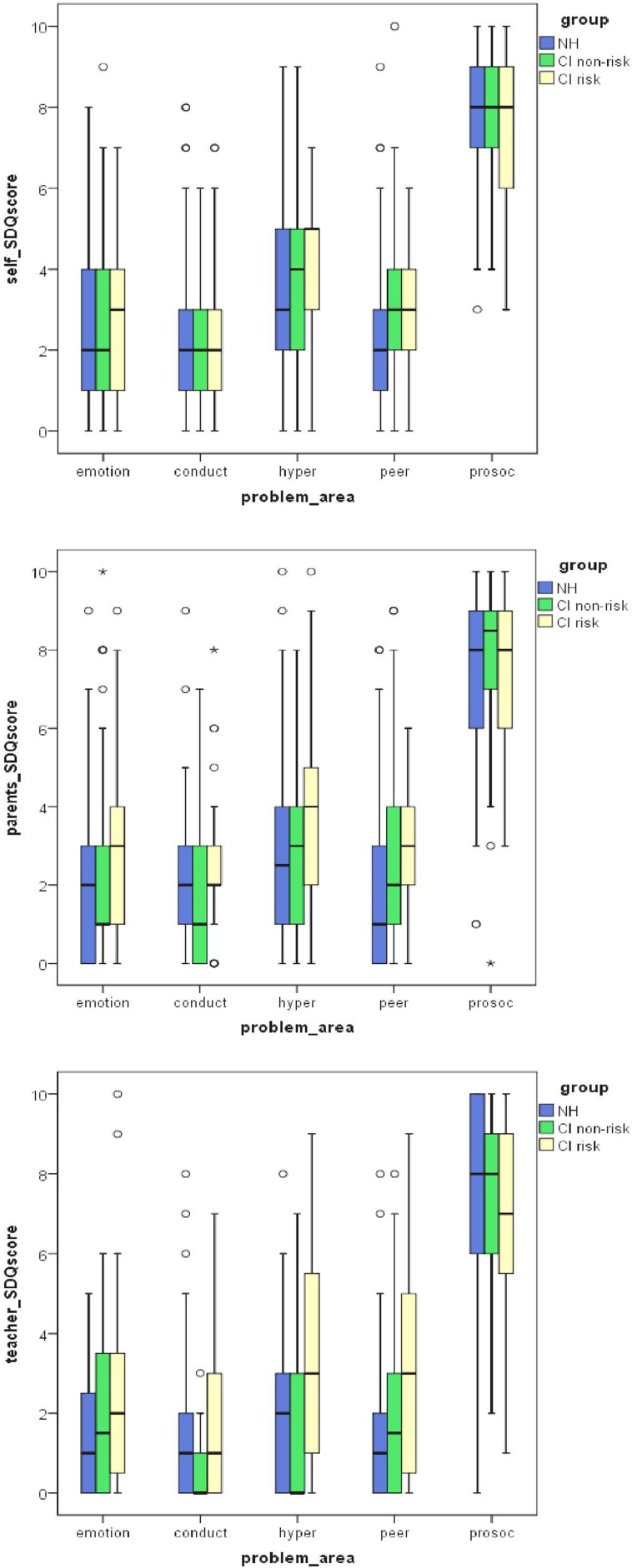
**Box-plots of SDQ self- parent- and teacher-ratings for emotional symptoms, conduct problems, hyperactivity and peer problems as well as pro-social behavior in normally hearing (NH) adolescents and CI adolescents with (risk) and without (non-risk) additional handicaps**. CI adolescents without additional handicaps differ from NH adolescents only in their self-rated peer problems. CI adolescents with additional risks differ from NH adolescents not only in peer problems, but also in hyperactivity and from CI adolescents without additional risks in conduct problems. Higher scores indicate more problems. Circles (°) and asterisks (^*^) indicate extreme cases and outliers.

In a next step, the total CI group was split into a risk group, including 35 “risk cases” and 11 cases, who could not be assigned, as well as a non-risk group, including all 94 CI adolescents without additional risks. The risk-group and the non-risk group did not differ with respect to age, gender or social background. To evaluate differences in mental health difficulties between risk-group and non-risk group on the one hand as well as between both groups and the non-impaired NH adolescents on the other hand, the multivariate ANOVAs were repeated with group (CI risk-group, CI non-risk group) as independent variable. The results are displayed in Figure 1. As before, highly significant multivariate group differences were observed for self-, parents- and teacher ratings (all *F* > 2.94, all *p* < 0.01).

*Post-hoc* Tukey tests revealed that among CI adolescents risk group and non-risk group differed significantly in parent- and teacher-rated conduct (CP), and hyperactivity problems (HA) (all *p*_*posthoc*_ < 0.05), but not in emotional symptoms (ES) and peer problems (PP) (all *p*_*posthoc*_ > 0.06). Thereby, conduct problems (CP) did not differ between any CI group (risk- group and non-risk group) and NH group, whereas hyperactivity (HA) did only differ between risk- group and NH group, but not between non-risk group and NH group normal. No differences in emotional, conduct or hyperactivity problems were observed between the three groups for self-ratings. For parent- and teacher ratings only the risk-group differed from the NH group in peer problems (PP) (both *p*_*posthoc*_ < 0.05). For self-ratings both CI risk-group and CI non-risk group differed from the NH group in peer problems (PP; both *p*_*posthoc*_ < 0.05). Prosocial behavior (PBS) ratings did not differ between groups in any rater (compare Figure 1).

### Cluster analyses of SDQ results and distribution of adolescents with CI (*n* = 140) and the matched group of NH adolescents (*n* = 140)

Cluster analyses over the 5 ratings of self, parents and teachers, each, revealed two clusters, discriminating significantly between participants with high and low problem scores (all |*t*| > 2.84, all *p* < 0.005). The distribution of CI adolescents and NH adolescents did not differ between clusters in the analysis of parent- (52% CI, 43% NH in Cluster 1, *X*^2^ = 1.51, *p* = 0.22) and self-ratings (46% CI, 36% NH in Cluster 1, *X*^2^ = 2.64, *p* = 0.10). However, the distribution of CI adolescents and NH adolescents did differ significantly between clusters in the analysis of teacher ratings (34% CI, 12% NH in Cluster 1, *X*^2^ = 6.56, *p* = 0.01). CI adolescents were more frequent in the high problem cluster than in the low problem cluster.

When the CI group was split into risk group and non-riskgroup, a significantly higher proportion of risk cases than non-risk cases or NH adolescents was found in the high problem cluster for self- (61% risk, 38% non-risk, 36% NH in Cluster 1, *X*^2^ = 8.43, *p* = 0.02), parent- (71% risk, 43% non-risk, 43% NH in Cluster 1, *X*^2^ = 10.71, *p* = 0.005), and teacher-ratings (37% risk, 33% non-risk, 12% NH in Cluster 1, *X*^2^ = 6.64, *p* = 0.04).

### SDQ results and the role of hearing (correlations and T-tests) study group (*n* = 140)

To investigate the role of hearing for mental health problems we used the following hearing variables: (i) (even) minimal benefit of hearing aid prior to implant, (ii) age at implantation of the first CI, (iii) monaural, binaural implantation, (iv) speech discrimination (monosyllables, 65 dB), (v) ability to hear and to understand speech in noise and (vi) aided thresholds or functional gain of CI (means of 0.5 kHz; 1 kHz, 2 kHz, 4 kHz), see Table 2. Because of missing data the age at first fitting of HA was not taken into account.

Age at implantation of the first CI (ii) monaural, binaural implantation (iii) and comprehension of monosyllables (iv) did not significantly relate to any SDQ outcome (all *p* > 0.05).

Peer problems (PP), as assessed by self ratings, showed significant negative correlations with minimal benefit of hearing aids. The higher the benefit of hearing aid prior to implant, the lower were the PP scores (*r* = −0.20, *p* < 0.05).

On the other hand, the Total Difficulty Score (TDS) and peer problems (PP), as assessed by teacher ratings, revealed a significant positive correlation with minimal benefit of hearing aids. The higher the benefit of hearing aids prior to implantation, the higher were the TDS (*r* = 0.27, *p* < 0.05) and PP scores (*r* = 0.33, *p* < 0.05).

Furthermore, the TDS and hyperactivity (HA), as assessed by teacher ratings correlated significantly negative with aided thresholds. The lower the aided thresholds, the higher were the TDS (*r* = −0.40, *p* < 0.05) and HA scores (*r* = −0.37, *p* < 0.05).

To investigate possible associations between the ability to hear/to understand speech or speech intelligibility in noise (see Table 2) and SDQ outcomes, we established two groups. CI adolescents who were able to hear and to understand in noise (*n* = 84) reached significantly lower TDS scores (*t* = −2.23, *p* < 0.05) as assessed by parent ratings, than CI adolescents who were constricted regarding understanding in noise (*n* = 30).

## Discussion

In a multi-center study we investigated 140 adolescents with hearing loss and CI(s) and 140 normally hearing peers, matched for age, gender and social background.

The agreement between the SDQ informants in the CI group was found to be higher than in earlier studies on mental health of young CI-users (Huber and Kipman, 2011; Anmyr et al., 2012). A high inter-informant agreement demonstrates a high validity of the SDQ results (Becker et al., 2004a,b) and a high predictive value for psychiatric diagnoses (ICD 10^16^, or DSM 5 (American Psychiatric Association, 2013). In the hearing group the agreement between the SDQ informant's parents and teachers was only low. Previous studies among healthy participants showed a higher inter-rater correlation, see Stone et al. (2010). Therefore, self-, parent-, and teacher ratings were compared separately between groups.

The CI group as a whole showed significantly more peer problems (PP) compared to the hearing group (all raters). The differences in the SDQ Total Difficulty Score (TDS), emotional symptoms (ES), conduct problems (CP), inattention-hyperactivity (HA), and pro-social behavior (PBS) were not significant.

When the CI group was split into a risk group (CI users with indications for additional handicaps and non-classifiable persons) and a non-risk group (CI users without any additional handicaps), increased peer problems compared to NH adolescents were observed in both subgroups according to self-ratings, but only in the risk group according to parent- and teacher-ratings. Whereas self-ratings did not indicate any differences between risk and non-risk group, parent- and teacher-ratings indicated additional mental health problems in the risk group compared to NH adolescents (hyperactivity) and the non-risk group (conduct problems). Cluster analyses of the SDQ results (emotional symptoms, conduct problems, hyperactivity, peer problems, prosocial behavior) confirmed that the distribution of persons with high problem scores was comparable for the NH and the non-risk group, but elevated among the risk group. Note however, that the teacher ratings (independent of the type of school) led to somewhat contrary results in the cluster analyses. As it was one limitation of the present study that only a minority of teachers participated, our further interpretations will focus mainly on the results of self- and parent-ratings. The phenomen of low teacher participation has also been reported in other studies (Keilmann and Reutter, 2014). We assume that particularly the engaged teachers participated in the study, those, who were motivated to help their pupils and work closely with the parents. They may however, not be entirely representative of all teachers in the study group.

In summary, our results indicate that despite their self-rated peer problems, the prevalence of mental health problems does not differ between NH adolescents and adolescents with CI, if they have no additional handicaps. However, as hypothesized, adolescents with CI, who do have additional handicaps show more problems compared to both NH adolescents and non-risk adolescents with CI.

The higher inter-rater agreement in the CI group compared to the NH group may indicate higher problem awareness in parents and teachers of CI adolescents. Higher awareness may also explain, why peer problems were only perceived by the adolescents themselves in the non-risk group, but by all three raters in the risk group. In the case of additional handicaps, care-givers may be more alert to signs of problems on the one hand and problems more obvious on the other hand. Thus, they become noticed not only by the adolescents themselves, but also by parents and teachers.

The result of additional mental health problems in the risk group correspond to the results of previous studies about mental health problems and disorders of young persons with a hearing loss and without CI (Hindley et al., 1994; Vostanis et al., 1997; Van Eldik, 2005; Van Gent et al., 2007, 2012; Fellinger et al., 2008; Landsberger et al., 2013). However, they also correspond to the results of previous studies about mental health problems and disorders of young NH persons with learning intellectual disabilities or learning disorders (Carvill, 2001; Barkauskiene and Bieliauskaite, 2002; Dekker et al., 2002; Leask et al., 2002; Glazebrook et al., 2003; Hemmings et al., 2006; Kaptein et al., 2008; Emerson et al., 2010; Backenson et al., 2013) visual impairment (Carvill, 2001) and problems in language and speech (e.g., Helland et al., 2014; Charman et al., 2015). For example, in an Australian study (Emerson et al., 2010), a nationally representative sample of NH children (age 6/7 years) was investigated. SDQ parent-ratings indicated that children with intellectual disabilities and children with borderline intellectual functioning “showed significantly higher rates of possible mental health problems” compared to “typically developed” children.

The diverse outcomes of CI users with and without additional handicaps indicate that it is important to differentiate between these two subgroups. This may explain variability between previous studies not controlling for additional risk factors (Hindley et al., 1994; Vostanis et al., 1997; Van Eldik, 2005; Van Gent et al., 2007, 2012; Fellinger et al., 2008; Landsberger et al., 2013). The presence of a disabling physical health condition may increase the vulnerability for mental health problems in young persons with a hearing loss with and without CI.

Concerning peer problems, earlier studies on children and adolescents with CI (Huber and Kipman, 2011; Martin et al., 2011) and with hearing loss, but without CIs (Wolters et al., 2011) yielded similar results. According to the results of a small study (*n* = 10), peer problems of children with CI begin very early at the age of 5–6 years (Martin et al., 2011). In adolescence the reverse of peer problems is a state of acceptance and popularity in the group, based on a successful interaction with peers. Peer problems can be associated with being bullied and teased, which seem to be more often the case for children and adolescents with hearing loss than for NH adolescents (Fellinger et al., 2008; Van Gent et al., 2012). These problems may in part stem from the CI adolescents being perceived as different by their peers.

However, Wolters et al. (2011) found the following skills and attributes to be essential to prevent peer problems of adolescents with a hearing loss (without CI): strategic and pragmatic communicative skills, social skills (prosocial behavior and the absence of antisocial behavior) and personality (extraversion, agreeableness). Since no differences between the groups were observed in prosocial behavior, it is likely that the higher rates of peer problems in CI adolescents have their roots in distinct communication problems, which impede their interaction with peers. According to earlier studies, young persons with a hearing loss without CI (Fellinger et al., 2008; Barker et al., 2009; Kushalnagar et al., 2011), but also with CI (Ramirez-Inscoe and Moore, 2011) have problems with communicative skills. Communication problems may be based on language problems, partly determined by intellectual disability, borderline intellectual functiononing (Holt and Kirk, 2005) or a malformed cochlea. However, according to self-ratings peer problems are also prevalent in CI adolescents without these additional handicaps.

It seems plausible that despite many years of hearing experience via the CI and hearing aids, communication problems stem from the hearing impairment in adolescents with CI.

Therefore, we assessed possible relations between hearing variables like age at CI and mental health outcomes. While contrary to our expectations, the age at first CI, the duration of CI use as well as the audiological results were not related to mental health problems (but see teacher ratings), the results indicate that particularly the ability to hear and to understand (speech intelligibility) in noise may be important. Difficulties of young CI users to understand people in noisy environments, such as schools, may induce social isolation and mental health problems, particularly peer problems, in the long term. Since the study centers had used different speech discrimination tests, we used the dichotomic assessment of the audiologists (Table 2) to inform about the ability to understand in noise. Furthermore, the mental health of adolescent CI users was related to the minimal benefit of hearing aids prior to the cochlear implant in the first years of life. Illg et al. (2013) found significant differences in speech comprehension depending on the duration of hearing aid use before second implantation in adolescents with sequential bilateral CI. Therefore, all patients should be encouraged to continue wearing their contralateral hearing aid in order to maintain afferent neural activity. Minimal benefit of hearing aids in the first years of life may indicate better speech understanding later on.

In the case of NH children, missing acceptance by peers is accompanied by anxiety in the long term (Grover et al., 2007). Being bullied is associated with low self-esteem and emotional problems in the long term (Bond et al., 2001; Woods et al., 2009).

Thus, parents, teachers, and clinicians should be aware that adolescents with CI, especially those with communication problems and those with additional special needs tend to have more problems with their peers. However, the self-perceived peer problems in adolescents with CI, who do not have additional handicaps, should not be dismissed.

## Conclusion

In summary, the results of this multi-center study indicate that mental health problems of adolescents with CI concern particularly the interaction with peers. Apart from that, and if there are no additional handicaps, the mental health (emotional, behavioral and social problems) of CI children is comparable to that of normally hearing peers. The benefit of hearing aids prior to implantation because of residual hearing in the first years of life and the ability to understand in noise was found to be protective against mental health problems. This multi-center study belongs to pioneer studies which inform about the mental health of adolescents with hearing loss, who are growing up with cochlear implants. To the best of our knowledge, this is the first study in this area including a large sample, a carefully matched control group and controlling for additional risk factors. The study also belongs to the first assessing the relationship between hearing variables (e.g., age at CI) and mental health problems of young CI users.

## Author contributions

MH was the leading investigator. She developed the proposal for the multi-center study, organized the funding and converted the centers to the cooperation. She was responsible for the data of the control group and played a leading role in the composing of this paper. As first author she is primarily accountable for all aspects of the work. TB—co-work in the conception and designing the study-monitoring parts of the sampling survey and data-acquisition -being co-author in writing parts of he manuscript (most notably “results”) -approvement oft the “end-version”- I agree to be accountable for all aspects of the work an I ensure that questions related to the accuracy or integrity of any part of the work are appropriately investigated and resolved. AI Substantial contributions to the acquisition of the patient data, and interpretation of data for the work; and Drafting parts of the paper; and Final approval of the version to be published; and Agreement to be accountable for all aspects of the work in ensuring that questions related to the accuracy or integrity of any part of the work are appropriately investigated and resolved. SiK gave substantial contributions to the acquisition, analysis, and interpretation of data for the work, to revising it critically for important intellectual content, to the final approval of the version to be published and her agreement to be accountable for all aspects of the work in ensuring that questions related to the accuracy or integrity of any part of the work are appropriately investigated and resolved. AG “I have contributed to the acquisition of data, to the revision of the work, to ensuring that the questions of the work are appropriately investigated, and to the approval of the version to be published.” LB provided substantial contributions to the design of the work; the acquisition and analysis of data for the work; and revised the important content critically and gave the final approval of the version to be published and agrees to be accountable for all aspects of the work in ensuring that questions related to the accuracy or integrity of any part of the work are appropriately investigated and resolved. StK- co-work in conception and designing the study-monitoring parts of the sample-recruiting and acquisition of data-being co-author in writing parts of the manuscript (most notably “discussion”)- improvement of the “end-version”- I agree to be accountable for all aspects of the work an I ensure that questions related to the accuracy or integrity of any part of the work are appropriately investigated and resolved. AN gave substantial contributions to the conception of the work, to the acquisition of data for the work, regarding critical revisions of the manuscript, and regarding the final approval of the version to be published. GR contributed substantially to the conception of the work. He revised the draft version of the paper critically and approved the final version. He is accountable for all aspects of the work. AB contributed to the conception of the work, counseled in basic questions about the SDQ, reviewed substantially the draft version and approved the final version of the paper. He is accountable for all aspects of the work. AK provided substantial contributions to the conception and design of the work; the interpretation of data for the work; and drafted the work and gave a final approval of the version to be published and agrees to be accountable for all aspects of the work in ensuring that questions related to the accuracy or integrity of any part of the work are appropriately investigated and resolved.

### Conflict of interest statement

The authors report institutional, but no personal grants from the research fund of Cochlear. The authors declare that the research was conducted in the absence of any commercial or financial relationships that could be construed as a potential conflict of interest.

## Footnotes

^1^Mental health problems are described on the focus of behavior as emotional, behavioral and social problems. Another form of mental health is HRQoL (health related quality of life), with the focus on the (subjective) personal experience. Mental health disorders have to fulfill nosological criteria as described in ICD 10 (World Health Organization 2010). International Statistical Classification of Diseases—ICD 10. http://apps.who.int/classifications/icd10/browse/2010/en (assessed 19.2.2014). or DSM 5 (American Psychiatric Association, 2013).

^2^“Deaf” and “hard-of-hearing” according to Dammeyer (2010).

^3^SDQ Strengths and Difficulties Questionnaire (Goodman, 1997).

^4^(a) Child Depression Inventory (self) (Kovacs, 1985) (self report).

^5^(b) Questionnaire (1) out of the study of Rieffe et al. (2004), Questionaire (2) out of the study of Wright et al. (2010) (both self reports).

^6^(c) Fear Survey Schedule for Children -Revised, shortened version (self report) (Ollendick, 1983).

^7^(d) Homemade questionnaire (self report).

^8^(e) Child Symptom Inventory (parents) (Gadow and Sprafkin, 1994).

^9^(f) Self report Instrument for reactive and proactice aggression (Rieffe et al., unpublished paper).

^10^(g) Delinquency Questionnaire (self report) (Baerveldt et al., 2003).

^11^(h) Psychopathy Screening Device (parents) (Frick et al., 1994).

^12^(i) The authors used the scales “attention deficit hyperactivity disorder” and “oppositional defiant behavior” of CSI-4 “The Child Symptom Inventory” (parents) (Gadow and Sprafkin, 1994).

^13^Thirty eight did not participate, as many girls as boys. 21 families excused themselves because of time reason, 17 did not react to the invitation per mail.

^14^International Labor Office (1990). ISCO-88: International Standard Classification of Occupations. Genf: ILO, International Labor Office. http://www.warwick.ac.uk/ier/ (assessed 19.2.2014).

^15^Youth in mind (2014). SDQ Information for researchers and professionals about the Strengths and Difficulties Questionnaire. http://www.sdqinfo.com (assessed 29.9.2014).

^16^World Health Organization 2010. International Statistical Classification of Diseases—ICD 10. http://apps.who.int/classifications/icd10/browse/2010/en (assessed 19.2.2014).
